# Toxic Peptides from the Mexican Scorpion *Centruroides villegasi*: Chemical Structure and Evaluation of Recognition by Human Single-Chain Antibodies

**DOI:** 10.3390/toxins16070301

**Published:** 2024-07-01

**Authors:** Lidia Riaño-Umbarila, Timoteo Olamendi-Portugal, José Alberto Romero-Moreno, Gustavo Delgado-Prudencio, Fernando Z. Zamudio, Baltazar Becerril, Lourival D. Possani

**Affiliations:** 1 Investigadora por México, CONAHCYT, Mexico City 03940, Mexico; lidia.riano@ibt.unam.mx; 2 Departamento de Medicina Molecular y Bioprocesos, Instituto de Biotecnología, Universidad Nacional Autónoma de México, Cuernavaca 62210, Mexico; timoteo.olamendi@ibt.unam.mx (T.O.-P.); jose.romero@ibt.unam.mx (J.A.R.-M.); gustavo.delgado@ibt.unam.mx (G.D.-P.); fernando.zamudio@ibt.unam.mx (F.Z.Z.)

**Keywords:** *C. villegasi*, single-chain fragment variable (scFv), scorpion toxins, recognition

## Abstract

Alternative recombinant sources of antivenoms have been successfully generated. The application of such strategies requires the characterization of the venoms for the development of specific neutralizing molecules against the toxic components. Five toxic peptides to mammals from the Mexican scorpion *Centruroides villegasi* were isolated by chromatographic procedures by means of gel filtration on Sephadex G-50, followed by ion-exchange columns on carboxy-methyl-cellulose (CMC) resins and finally purified by high-performance chromatography (HPLC) columns. Their primary structures were determined by Edman degradation. They contain 66 amino acids and are maintained well packed by four disulfide bridges, with molecular mass from 7511.3 to 7750.1 Da. They are all relatively toxic and deadly to mice and show high sequence identity with known peptides that are specific modifiers of the gating mechanisms of Na^+^ ion channels of type beta-toxin (β-ScTx). They were named Cv1 to Cv5 and used to test their recognition by single-chain variable fragments (scFv) of antibodies, using surface plasmon resonance. Three different scFvs generated in our laboratory (10FG2, HV, LR) were tested for recognizing the various new peptides described here, paving the way for the development of a novel type of scorpion antivenom.

## 1. Introduction

Mexico is a country with a rich biodiversity of scorpion species and occupies the first place in the world for incidence of stings to humans (circa 300,000 a year) [1,2]. Fortunately, there are antivenoms produced in horses that have helped to keep deadly cases to a small number [3]. However, commercially available antivenoms are prepared to act against the venom of four scorpion species of the genus *Centruroides*. Antibodies from horse plasma are digested with pepsin, resulting in antibody fragments of the F(ab’)_2_ type. These fragments of antibodies are not biochemically and individually identified. The administration of the antivenoms can eventually produce side effects in some patients [4]. In addition, the homogeneity of the horse F(ab’)_2_ used is not well known, and antibodies against irrelevant proteins/peptides of the venom are also included in available antivenoms [5]. Additionally, they have not been evaluated against all species of harmful Mexican scorpions, since many of these species have not yet been described and characterized.

Our research group has dedicated effort to identifying the species of scorpion most dangerous to humans among the 310 different known species [6] of scorpions in the country. The list of known dangerous scorpions contains more than 20 different species [7]. An important contribution of our group consisted of the identification of distinct medically important peptides due to their abundance and degree of toxicity to humans. This list of deadly toxins is small considering that hundreds of different components were identified in the venom of the scorpions under study [8]. We have shown that only about two to four different peptides in each species of the genus *Centruroides* have significant toxicity to humans [9,10,11,12]. 

The most important peptides are those that recognize the voltage-gated sodium channels of excitable cells [13,14,15,16]. Their structures are similar in terms of the number of amino acids in the sequence and the three-dimensional structures [17]. The differences between some amino acids in the primary structure of the toxins determine the level of toxicity and their specificity to sodium channels. Similarly, if these toxin variations are found in the epitopes, these changes determine that an antibody will neutralize some toxins but not necessarily all of them.

The rationale followed was that if only a few peptides of each species of scorpion need to be neutralized by a potential antivenom, then a well-characterized antibody fragment at the level of immunological recognition and neutralizing capacity would be sufficient for neutralization of the whole venom. In addition, if the fragment of protecting antibody could be reduced to a low molecular mass, like the single-chain fragment variable (scFv) [18], and could be produced by recombinant DNA of microorganisms instead of horses, production could be modernized. If the initial antibodies were of human origin and matured against scorpion toxins, this could create a better antivenom. This is the essence of our study and our long-term objective. 

Following this rationale, we have dedicated many years to this project and have found interesting results, further discussed later in this manuscript. To fully develop this idea, we first needed to identify the dangerous species, purify their abundant and toxic components, determine their structure, and assay scFv fragments potentially capable of neutralizing the toxic effect.

This communication describes the purification and characterization of five new toxic peptides from one of the dangerous species found in the State of Guerrero, Mexico [19]. The potential neutralization of three different types of human scFv fragments developed by our group was assessed using surface plasmon resonance (SPR).

## 2. Results

### 2.1. Purification and Sequencing of Toxic Peptides

**Figure 1:**
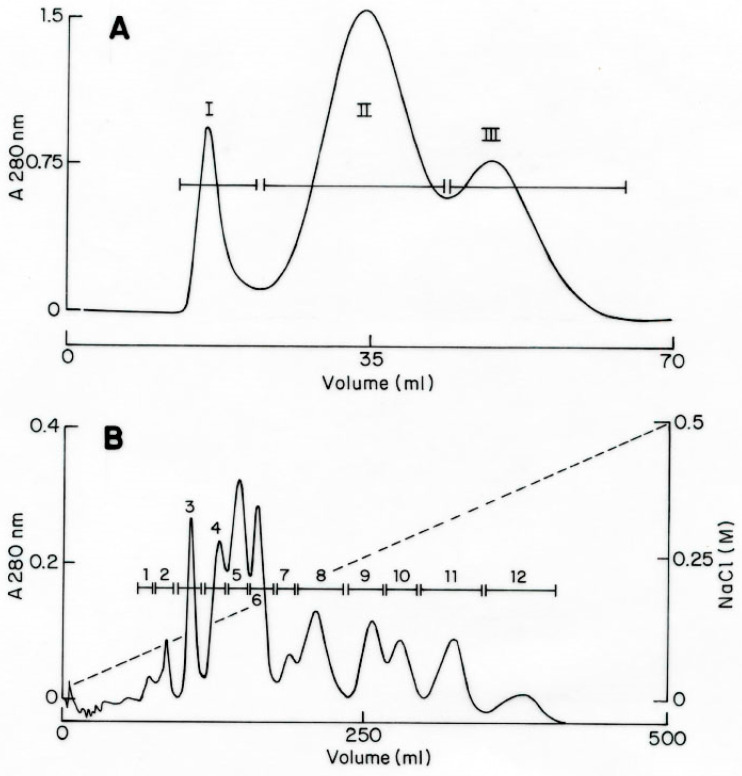
Purification of toxic peptides: (**A**) Sephadex G-50 separation of 33 mg of soluble venom from *C. villegasi*. Column dimensions: (1 × 50 cm). Equilibrated and run at 1 mL/min in the presence of 20 mM of ammonium acetate buffer, pH 4.7. (**B**) Separation of F-II of A (24 mg) through a CMC column (1 × 5 cm) run in 20 mM of ammonium acetate buffer, pH 4.7, at 0.5 mL/min and eluted with a gradient of NaCl from 0 to 0.5 M for 1000 min.

**Figure 2:**
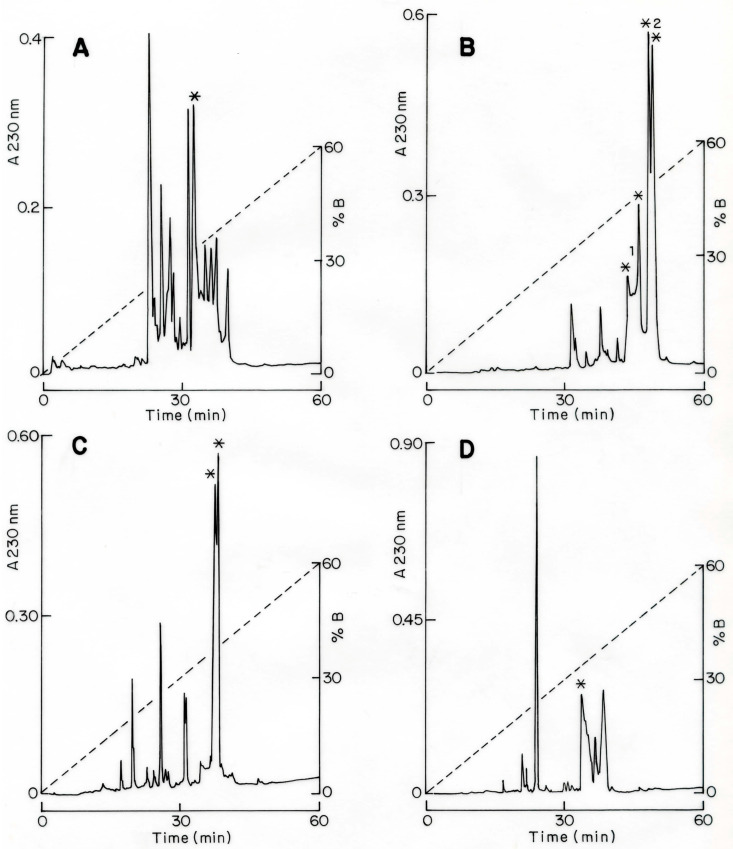
Purification of peptides by HPLC (**A**–**D**). Separation of fractions II-8 to II-11 on a C18 reverse-phase HPLC column with a gradient prepared with solution A, containing 0.12% TFA in water to 60% of solution B in 0.10% TFA, run for 60 min. All peptides indicated by asterisks were individually sequenced. Overlapping the amino acid sequences obtained after enzymatic hydrolysis with Asp-N endopeptidase (see Supplementary Material) of each peptide allowed the determination of the full sequences, as indicated in Figure 3.

**Figure 3:**
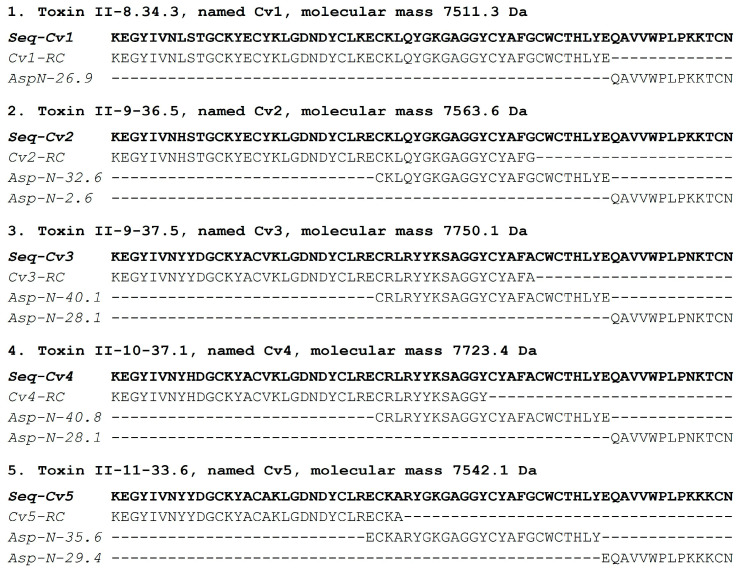
Amino acid sequence of pure toxins (in bold type) obtained through fragmentation processes.

The soluble venom of the species *Centruroides villegasi* (hereafter abbreviated to *C. villegasi*) was initially separated using a Sephadex G-50 column, which clearly showed three different fractions (I to III), as shown in Figure 1A. The recovery of fractions was quantified. Toxicity tests of samples of the column into mice showed that only fraction II was lethal. Fraction II was applied to a CMC column separating at least 12 sub-fractions (Figure 1B). Fractions II-8 to II-11 were further separated by HPLC chromatography. Peptides isolated on time II-8-34.3 (Figure 2A), II-9-36.5 (Figure 2B), II-9-37.5 (Figure 2B), II-10-37.1 (Figure 2C), and II-11-33.6 (Figure 2D) were sequenced. Each one of the pure peptides was separately sequenced by Edman degradation in its native format and after reduction and alkylation. The carboxymethylated format was used to confirm the position of the cysteines. In addition, a sample of the reduced and alkylated peptides was cleaved by a proteolytic enzyme (aspartic N endopeptidase). The peptides of the digestion with the Asp-N enzyme were separated by HPLC, as shown Figure S1. Each sub-peptide obtained after enzymatic hydrolysis was subjected to Edman degradation, and the amino acid sequences were used to overlap the full sequence. For all five new peptides, overlapping the identified sub-peptides permitted obtaining the full sequence. The results obtained are included in Figure 3. They were named Cv1 to Cv5, abbreviated from *C. villegasi* (Cv) toxins 1 to 5, according to what is indicated in the figure. The sequences were deposited in UniProt Knowledgebase with the following accession numbers: C0HMB8 for Cv1, C0HMB9 for Cv2, C0HMC0 for Cv3, C0HMC1 for Cv4 and C0HMC2 for Cv5. The molecular masses of the toxins (theoretically calculated and experimentally determined) were within an acceptable experimental error range (Cv1 7511.6 found 7511.3 Da; Cv2 7563.6, found 7563.6 Da; Cv3 7749.8 found 7750.1 Da; Cv4 7723.8, found 7723.4 Da; Cv5 7542.7 found 7542.1 Da).

### 2.2. Venom Toxicity and Amount of Pure Toxins

It is important to mention that previously, the fresh and soluble venom of collected scorpions was used for the determination of the media lethal dose (LD_50_) in CD1 mice. Scorpions were milked for venom by electric stimulation on the telson, and the soluble venom was used for the determination of LD_50_. A value of 12.2 µg/20 g body weight of mice was initially obtained, as published elsewhere [20]. The toxicity of the venom is due to the presence of different toxic components. From chromatographic purification processes, it is possible to estimate the relative abundance of these components. The amount of Cv1 toxin was 0.8% of the soluble venom and proved to be slightly toxic to mice. The amount of peptide used for toxicity experiments in mice was 3.3 µg per animal, in all cases reported here. Toxin Cv2 corresponded to 1.7% of the venom and is also slightly toxic to mice. Toxin Cv3 was 3.0% of the soluble venom and with strong signs of intoxication in mice. Toxin Cv4 accounted for 2.1% of the venom and was also very toxic. Cv5 corresponded to 1.8% of the soluble venom and was moderately toxic to mice (see Section 5 for description of toxicity or lethality tests). Since these are new peptide sequences reported for the first time from *C. villegasi*, it was important to contrast with previously reported toxin sequences affecting mammalian sodium channels through phylogenetic analysis. A characterization of the interaction of the antibody fragments previously generated in the group was also performed.

### 2.3. Phylogenetic Tree

**Figure 4:**
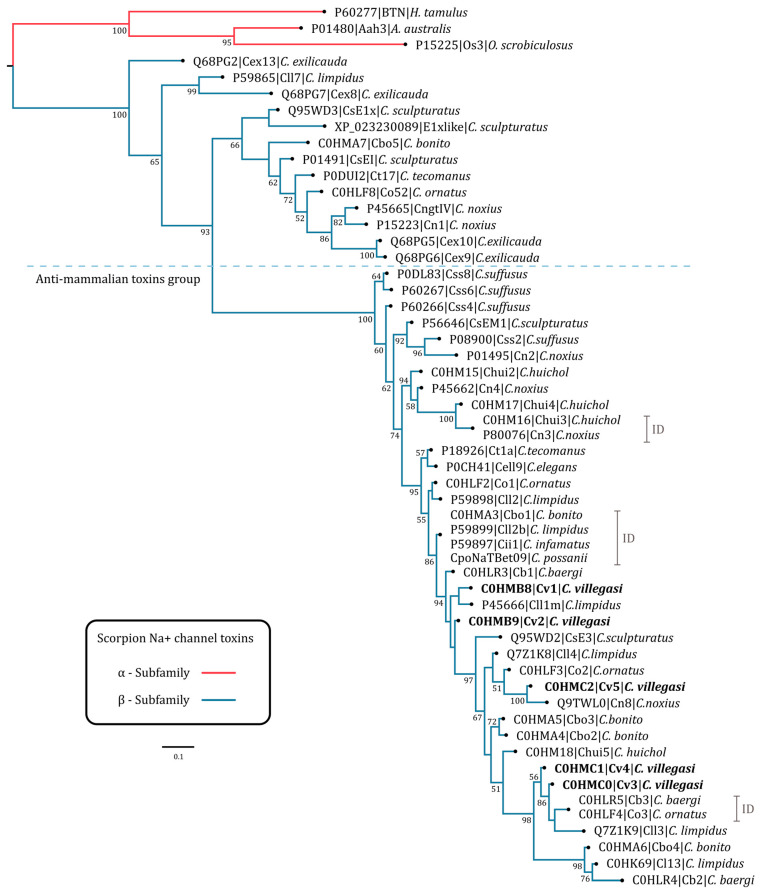
Topology of the Bayesian phylogenetic tree of *C. villegasi* toxins and other related NaScTx. ID indicates identical sequences found in other scorpions. Numbers under the nodes indicate percentage posterior probability values greater than 50. The scale bar represents the number of amino acid substitutions per site. Sequence names are composed of the UniProt or NCBI accession code or assigned in the original publication, followed by the toxin name and the scorpion species name. Three α-NaScTx (BTN, Aah3 and Os3) were used as outgroups and to root the tree. The dotted line dividing the tree separates the anti-mammalian group of toxins into basal clades.

Phylogenetic analysis showed that the five toxins of *C. villegasi* are closely related to Na^+^ toxins of the beta subfamily (β-NaScTx) of various scorpions of the genus *Centruroides*. The topology of the phylogenetic tree shows that *C. villegasi* toxins are located in one of the major clades well supported by a posterior probability percentage value of 93; in this clade, toxins with anti-mammalian activity with lengths between 65 and 67 amino acids are clustered (Figure 4 and Figure S2A). Toxins of the basal clades are shorter, with a length of 63 to 65 amino acids, and they exhibit diverse activities, e.g., the toxin CsEl (UniProtID: P01491) from *C. sculpturatus*, affects chicken and frog carcasses but not mammalian ones [21,22]. There are also toxins with no known function, e.g., Co52 (UniProtID C0HLF8) from *C. ornatus* [9], Ct17 (UniProtID: P0DUI2) from *C. tecomanus* [23], and Cbo5 (UniProtID: C0HMA7) from *C. bonito* [12], among others. The five *C. villegasi* toxins share between 82 and 97% amino acidic identity (Figure 4 and Figure S2). The toxin phylogenetically closest to Cv1 is the amidated toxin Cll1m (UniProtID: P45666) from *C. limpidus*, with 95% amino acid identity. Although Cv2 shares a 97% percent identity with Cv1, phylogenetic analysis recurrently positions Cv2 in a separate sub-clade of Cv1 (Figure 4); this clade is weakly supported (with a posterior percent probability less than 50), probably due to a lack of known orthologs in the databases. Toxin Cv5 clusters with the anti-mammalian toxins Cn8 (UniProtID: Q9TWL0) from *C. noxius* [24], Co2 (UniProtID: C0HLF3) from *C. ornatus* [9], and Cll4 (UniProtID: Q7Z1K8) from *C. limpidus*, with amino acid identity percentages of 98, 98, and 92%, respectively (Figure 4 and Figure S2). Toxins Cv4 and Cv3 (ordered by their position in the phylogenetic tree) share 98% amino acid identity, with Cv3 differing by a single amino acid at position 9 with respect to Cv4 (Y9H) (Figure S2). The toxins phylogenetically closest to Cv4 and Cv3 are the identical toxins Cb3 (UniProtID: C0HLR5) from *C. baergi* [10] and Co3 (UniProtID: C0HLF4) from *C. ornatus* [9] (Figure 4) and Cll3 (UniProtID: Q7Z1K9) of *C. limpidus*, with which Cv4 shares identity percentages of 95 and 91%, respectively (Figure S2).

### 2.4. Surface Plasmon Resonance Analysis

**Figure 5:**
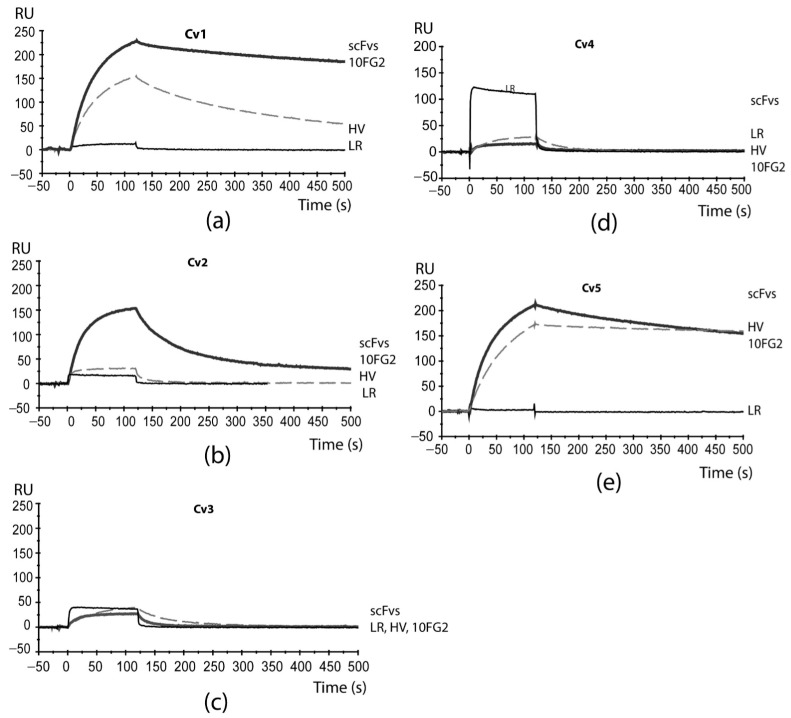
Sensorgrams of the interaction of scFvs and *C. villegasi* toxins. The scFvs were applied a 100 nM concentration in a flow rate of 50 µL/min at a temperature of 25 °C. (**a**) Assays against Cv1 toxin, (**b**) assays against Cv2, (**c**) assays against Cv3, (**d**) assays against Cv4, and (**e**) assays against Cv5.

Each of the toxins were individually immobilized on the CM5 chip following the described protocol. Figure 5 shows the sensorgrams of each evaluation obtained in Biacore. The antibody fragments used are in the single-chain variable format (scFv), which corresponds to the variable domains of immunoglobulins linked by a 15 aa linker peptide. These fragments come from a human scFv library and were isolated against a toxin from the scorpion *C. noxius* (Cn2). Subsequently, these scFvs were matured against the Cn2 toxin and other scorpion toxins by directed evolution and protein engineering methods, giving rise to scFvs LR [25], 10FG2 [26] and HV [11].

The analyses show that the best interaction occurs between the Cv1 toxin and the scFv 10FG2, where a good association phase (corresponding zone between 0 and 120 s) and low dissociation (corresponding zone between 120 and 500 s) is observed (Figure 5a). We consider it to be a good interaction that remains over time. The scFv 10FG2 also has good interaction with the Cv5 toxin, as well as the scFv HV (derived from scFv 10FG2), which seems not to dissociate in the evaluated time (Figure 5e), which is also relevant. The scFv 10FG2 also recognizes the Cv2 toxin (Figure 5b); however, its interaction is weaker since it dissociates quickly from the toxin compared to the interactions of 10FG2 with Cv1 and Cv5. The toxins Cv3 and Cv4 are not yet well recognized by antibodies (Figure 5c,d). The scFv LR does not have the capacity to recognize any of the toxins of this venom.

To explain the recognition results of the scFvs, the relatedness of the sequence should be considered, since scFv HV was derived from scFv 10FG2, whereas scFv LR is from a different family. We must also consider that these two families of scFvs recognize different epitopes in the toxins [27]. The scFv 10FG2 can recognize and neutralize several toxins efficiently, such as Cn2 (from *C. noxius* venom) and Cll1 (from *C. limpidus* venom) [26] in a defined epitope (see Figure S3), while LR neutralizes Cn2 very well and does not recognize the Cll1 toxin. The sequence changes between the toxins Cn2 and Cll1 and the toxins of *C. villegasi* provide insights that important interactions with LR cannot be established with the new toxins. For example, salt bridges between the LR and the amino acid residues of D7 and E15 of Cn2 cannot be formed with *C. villegasi* toxins. In addition, steric hindrance is likely to occur due to aromatic residues on the side chains at position 17 of the toxins. Such is the case with Y17 of toxins Cv1 and Cv2, which does not allow good interaction with scFv LR (Figure 5a,b), whereas L17 of toxin Cn2 does allow this interaction. Another interesting result was that scFv 10FG2 exhibits good cross-reactivity with three of the *C. villegasi* venom toxins, so we sought to understand why the interaction between scFv10FG2 with Cv1, Cv2, and Cv5 occurs.

### 2.5. In Silico Analysis of the Toxin: scFv Structural Models Were Performed to Understand the Cross-Reactivity of scFv 10FG2 with the Three Toxins

**Figure 6:**
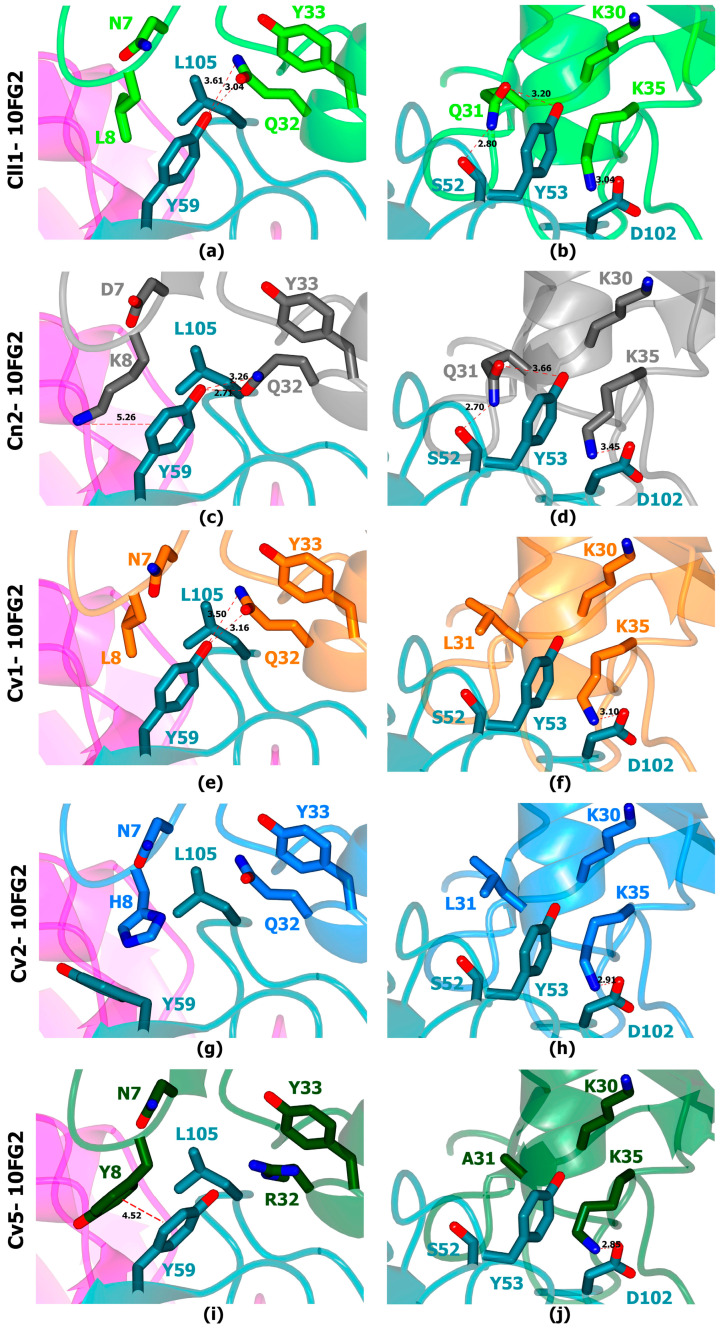
In silico analysis of scFv 10FG2 in interaction with the toxins Cll1 (light green) from *C. limpidus*, Cn2 (gray) from *C noxius*, Cv1 (orange), Cv2 (blue), and Cv5 (dark green) from *C. villegasi*. The main interactions at the interface between the different toxins and scFv 10FG2 are highlighted in the panels. Most interactions fall on the VH domain (turquoise) and to a lesser extent on the VL domain (magenta). In the case of Cll1 (**a**,**b**) and Cn2 (**c**,**d**) toxins, a greater number of interactions are observed with respect to those observed with Cv1 (**e**,**f**), Cv2 (**g**,**h**) and Cv5 (**i**,**j**) toxins. Among these are the hydrogen bridges of Q31 and Q32 of toxins Cll1 and Cn2 also formed in toxin Cv1 with Y59 VH of scFv 10FG2 in addition to the salt bridge of K35 of the toxins with D102 VH of scFv 10FG2.

The analyses showed that many of the contacts previously reported for the interaction of scFv 10FG2 with other similar toxins are maintained [26]. However, we found that some of the relevant residues in the interaction zone of scFv 10FG2 with its main targets Cll1 (Figure 6a,b) and Cn2 (Figure 6c,d) may not exist in the interaction between scFv 10FG2 and toxins Cv1 (Figure 6e,f), Cv2 (Figure 6g,h), and Cv5 (Figure 6i,j). It stands out that K35, of the three toxins, seems to keep the salt bridge interaction with D102VH of scFv 10FG2 (Figure 6f,h,j), as observed for toxins Cll1 (Figure 5b) and Cn2 (Figure 5d). On the other hand, the analysis suggests that the presence of non-polar aliphatic residues (L and A) at position 31 of the toxins does not allow the formation of two hydrogen bridges with the S52VH and Y53VH of scFv10FG2, which are formed with Q31 of the Cll1 and Cn2 toxins (Figure 6b,d). Another interaction site to highlight is residue 8 of the toxins. In the particular case of toxin Cv2, the presence of a His in this position could generate a steric hindrance with Y59 of scFv 10FG2, causing the reorientation of this residue and the loss of interactions that occur with Cv1 and Cv5 toxins. This could explain why Cv2 is still not well recognized by scFv 10FG2.

## 3. Discussion

The State of Guerrero is one of the places that harbors the greatest diversity of scorpion species of medical importance (*C. limpidus*, *C. balsasensis*, *C. meisei* and *C. bonito*, among others). Some communities are at risk because they are located in marginal areas, where they cohabit with dangerous species such as *C. villegasi* [28] in Chilapa, Guerrero. Therefore, it becomes important to investigate the toxic species in the country and look for alternatives to improve the quality of the treatment of scorpion stings.

The venom of *C. villegasi* is one of the most complex venoms studied to date, with five components of medical importance (abundance and toxic effects), which contrasts with most of the venoms of Mexican scorpions, which can have between two and four deadly toxins, [9,10,11,12], to mention some examples reported by our group. The five toxins described in this work correspond to 9.4% of the venom, and the toxicity previously reported by our group was 1 LD_50_ = 12.2 µg/mouse weighing 20 g, determined in the CD1 mouse strain. This value is close to the average, considering that the most toxic species is *C. noxius* with 1 LD_50_ 2.5 µg per 20 g mouse and the least toxic is *C. sculpturatus* with 1 LD_50_ 22.7 µg/20 g mouse [20].

We are surprised by the particularities of the venom toxins because the alignments showed that they are unique and have so far not been detected in other venoms. *C. villegasi* venom sequences maintain a high sequence identity among the toxins, and the phylogenetic analysis shows a closeness to other reported toxins; however, it is surprising that of these five, only Cv1, Cv2, and Cv5 share phylogenetic signals that group them with toxins that are recognized by the scFv 10FG2. The position of toxin Cv2 in the phylogenetic tree and its low branch support (<50) suggest that there may be a group of orthologs yet to be described in other species of the *Centruroides* genus. The topology of the phylogenetic tree also informs us of an ancestral relationship in the toxin sequences, with Cv1, Cv2, and Cv5 in ancestral clades compared to toxins Cv3 and Cv4, which cluster in a phylogenetically recent clade with other toxins that are difficult to recognize and/or neutralize with the evaluated scFvs.

After obtaining the main pure toxic components of this venom and evaluating the interaction with the scFvs, we know that the Cv1 and Cv5 toxins are well recognized and probably neutralized by the scFv 10FG2. Despite the possible loss of some contacts between scFv 10FG2 and toxins Cv1, Cv2 and Cv5, it can still be recognizing remarkably the toxins; this is explained by the versatility of scFv because it neutralizes with affinities between 10^−9^ o 10^−10^ M [26]. Toxins that are not yet well recognized or neutralized led us to consider the search for new scFvs. In particular, for the toxins Cv3 and Cv4 (the most toxic of the venoms) and even for Cv2, although the scFv 10FG2 recognizes them, it is still not sufficiently related to neutralize them.

Obtaining antibody fragments with broad cross-reactivity is of great importance in the development of new antivenoms, since a single antibody fragments can recognize and neutralize a large number of peptides that are similar in sequence and structure, as occurs in polyclonal commercial antivenoms [29,30,31]. As studied by our group, the scFv 10FG2 is an important antibody fragment due to its broad cross-reactivity and neutralizing capacity. It was obtained through several in vitro maturation processes, directing its recognition towards several toxins from Mexican scorpions. This work was no exception, since we determined that scFv 10FG2 recognizes three of the five components of medical importance in *C. villegasi* venom. Meanwhile, in the rest of the toxins (Cv3 and Cv4), the recognition of this antibody fragment is possibly affected by the Y34 of the Cv3 and Cv4 toxins, which could generate steric hindrance and loss of interactions with scFv 10FG2 residues.

Previous work has shown that it is possible to generate antibody fragments with phage display and directed evolution technologies [26,32,33]. These may be more efficient and safer because antibody fragments are generated only against toxins (which compromise human health). Thanks to the characterization of the venom and the evaluation of the toxic components, we identified the three toxins, which justify our work to obtain one or more new scFv(s) with the ability to neutralize them and achieve the neutralization of the whole venom.

## 4. Conclusions

Five peptides from the Mexican scorpion *C. villegasi* that are toxic to mammals were isolated, characterized, and evaluated based on their susceptibility to neutralization by scFvs generated in our group. *C. villegasi* is, as of now, the venom richest in toxic components within the *Centruroides* genus. Surface plasmon resonance analysis showed that scFv 10FG2 could recognize Cv1, Cv2, and Cv5 toxins. Cv5 toxin was also recognized by scFv HV. This interaction and the failure of scFvs to recognize toxins Cv3 and Cv4 were explained by the variations in the sequences with respect to those already neutralized.

## 5. Materials and Methods

### 5.1. Venom Source, Lethality Tests, and Purification Procedure

The scorpions were collected in the State of Guerrero with the permission of the Government agency (SEMARNAT, number SGPA/DGS/00367/22). Anaesthetized scorpions were electrically stimulated in the telson for venom collection. We used 20V DC in two pulses. The venom was recovered in distilled water and centrifuged for 15 min at 15,000× *g*. All of the soluble venom was lyophilized and kept at −20 °C until use.

Purification of peptides was performed by the procedure earlier described by our group [34]. It consists of gel filtration, followed by an ion-exchange column and high-performance liquid chromatography (HPLC). Initially, the venom was solubilized, and the concentration was estimated, assuming that one optical unit read in the spectrophotometer at 280 nm corresponds to 1 mg/mL of a mixture of soluble proteins. A total of 33 mg of soluble venom was separated by a Sephadex G-50 column in three fractions. Each fraction was evaluated in mice intraperitoneally to observe signs of intoxication or death. The toxic fraction (number II) was applied to carboxy-methylcellulose (CMC) beads, separating at least 12 sub-fractions. The ones toxic to mice (II-8 to II-11) were further separated by HPLC using a Waters model 1525 chromatographer (Milford, MA, USA) with a C18 reverse-phase column from Vydac (Hisperia, CA, USA), as earlier described. In the case of toxins, quantification was carried out with the theoretical molar extinction coefficient of the toxin, its molecular mass, and the absorbance value determined at 280 nm. We used the Beer–Lambert formula, C = A/ɛʃ, where C is the molar concentration, ɛ is the molar extinction coefficient, and ʃ is distance 1 cm [35]. The pure toxic components (Cv1 to Cv5) were used for further characterization (see above). 

Lethality tests of each pure toxin were conducted by injecting mice (CD1 strain) intraperitoneally with 3.3 µg of the corresponding toxin. The toxins were said to be “slightly toxic” when the mouse showed symptoms of piloerection, certain agitation, and nose rubbing. They were deemed “moderately toxic” when animals in addition showed some respiratory problems and slight salivation but recovered within a few hours after assay. Animals deemed “highly intoxicated” started jumping after the injection and showed profuse salivation and strong respiratory distress (respiring with an open snout), finally dying within 30 min after injection. It is worth mentioning that these physiological manifestations of intoxication can vary when the amount of toxin injected is higher than 3.3 µg. For these experiments, only two animals were used for each pure toxin, because our committee for the welfare of vertebrate animals recommends using a minimal number of animals.

### 5.2. Mass Spectrometry and Sequence Determination

Purified toxic peptides had their molecular mass determined by mass spectrometry by means of LCQFleet equipment from Thermo Fisher Scientific Inc. (San Jose, CA, USA). The amino acid sequences (primary structure) of the toxins were obtained by Edman degradation using an automatic machine, model PPSQ-31A/33A of the Shimadzu Protein Sequencer (Columbia, MD, USA). Initially, a sample of each purified peptide was subjected directly to the sequencer. A substantial segment of the N-terminal region was obtained by this procedure, but the cysteine residues were further determined using another sample of the same peptide reduced and alkylated with iodoacetic acid [34]. To obtain the entire sequence of the purified peptides, an additional sample of all the reduced and alkylated peptides (Cv1 to Cv5) was subjected to enzymatic digestion using the proteolytic enzyme aspartic acid endopeptidase (ASP-N) from the company Roche Diagnostics GmbH (Mannheim, Germany). The peptide/enzyme mixture was incubated at 37 °C overnight in a 50 mM sodium phosphate buffer, pH 8.2. The digested peptides were separated by HPLC with a gradient prepared with solution A, containing 0.12% trifluoracetic acid (TFA) in water to 60% of solution B in 0.10% TFA. The columns were run for each component for 60 min at a flow rate of 1 mL/min. Each eluted component was analyzed for purity by mass spectrometry analysis and sequenced by Edman degradation. The overlapping of the sequences of the digestion products and the verification of the completeness of the whole toxin sequence were confirmed by mass spectrometry determination of the pure native peptides and compared to the expected molecular mass based on the amino acid sequence that was experimentally determined.

### 5.3. Phylogenetic Analysis of the New Peptide-Toxins

The search for potential homologs of *C. villegasi* toxins was performed as described by [12]. The search for related toxins was performed with blastp searches with BLAST+ v2.15.0+ [36] in command line. All proteins from UniProtKB/Swiss-Prot (Release 2024_01) were downloaded and used as a database. The five *C. villegasi* sequences were used as query with a fixed evalue = 1 × 10^−16^. An additional blastp search was performed in the NCBI-nr online database. Phylogenetic tree construction was performed with the sequences of the mature chains of scorpion Na^+^-beta subfamily toxins (β-NaScTx) retrieved by blastp. Three Na^+^ toxins of the alpha subfamily (α-NaScTx) were added as an outgroup. The sequence set was aligned with MAFFT v7.490 using the progressive FFT-NS-2 method [37]. The percentages of amino acid identity and similarity between sequences were calculated with a “sequence manipulation suite” [38]. Construction of an initial phylogenetic tree was performed by Bayesian inference with MrBayes v3.2.7 [39]. The amino acid substitution model used was WAG [40]. The options, lset rates = gamma with prset aamodelpr = fixed, were set. Phylogenetic analysis was run for 1 × 10^7^ generations on eight strands with a sampling frequency every 1000 trees. Consensus tree construction was built by discarding the first 1 × 10^6^ trees (Burn-in). The initial tree was pruned by eliminating clades distant to the *C. villegasi* sequences. A new phylogenetic analysis by Bayesian inference was performed using the remaining β-NaScTx; the three α-NaScTx were retained as an outgroup.

### 5.4. Evaluation by Surface Plasmon Resonance (SPR) 

Human scFvs generated in our laboratory against Mexican scorpion toxins were tested with the five toxins from *C. villegasi* in a biosensor that detects molecular interactions in real time (Biacore X100, Uppsala, Sweden) [41]. Five sensor CM5 chips were prepared by covalent immobilization using the amino coupling kit, reaching binding levels of 200 RUs of Cv toxins 1–5 (1 RU corresponds to a change in surface concentration of approximately 1 pg/mm^2^). Each pure toxin (Cv1, Cv2, Cv3, Cv4, Cv5) was solubilized in 10 mM 2-(N-morpholino) ethane sulfonic acid (pH 6) and bound on cell 2. Cell 1 in the sensor (without bound) was used as a control. The scFvs LR, 10FG2, HV [11,25,26] (neutralizing human scFvs of scorpion toxins) were evaluated in the recognition to each toxin. The samples were solubilized in HBS-EP buffer (Biacore) at 100 nM concentration and 100 μL of the scFvs were injected over the chip at a flow rate of 50 μL/min at 25 °C, with a delay time of 500 s. The chip surfaces were regenerated with 10 mM HCl. The resulting sensorgrams were evaluated using BIA-evaluation software version 3.1.

### 5.5. In Silico Structural Analysis of Interactions of Cv1, Cv2, and Cv5 Toxins with scFv 10FG2 

In silico modeling of each complex was accomplished by homology modeling. The models were created through in silico inserted mutations on the crystallographic structure of the scFv RU1-Cn2 toxin–scFv LR ternary complex (PDB entry 4V1D) replacing the scFv RU1 structure by 10FG2. The percentage of identity between the scFvs RU1 and 10FG2 was 97.4% and between Cn2 and toxins Cv1, Cv2, and Cv5 was 81.8, 83.3, and 78.7%, respectively. The model was constructed using Coot’s standard rotamer library [42]. Subsequently, energy minimization was performed with YASARA software (Krieger et al., 2009). Determination of interactions in models was performed with PIC [43] and PISA [44] servers. Model schemes were created with the CCP4MG program and edited with Adobe Photoshop version 22.5.0.

## Data Availability

The sequences deposited in Uniprot Database will be available once the article is accepted and published.

## Supplementary Materials

The following supporting information can be downloaded at: https://www.mdpi.com/article/10.3390/toxins16070301/s1, Figure S1: HPLC separation of peptides after enzymatic cleavage; Figure S2: Comparison of *C. villegasi* toxin sequences; Figure S3: Analysis of possible interactions of the evaluated scFvs with *C. villegasi* toxins.
