# Supplementary material for: Toxic Peptides from the Mexican Scorpion Centruroides villegasi: Chemical Structure and Evaluation of Recognition by Human Single-Chain Antibodies
Source: Toxins (Basel). 2024 Jul 1;16(7):301. doi: 10.3390/toxins16070301 (PMC11280942; doi:10.3390/toxins16070301)
Supplement: Supplementary file 1 [file toxins-16-00301-s001.zip › toxins-3062533-supplementary.pdf]

Supplementary Material

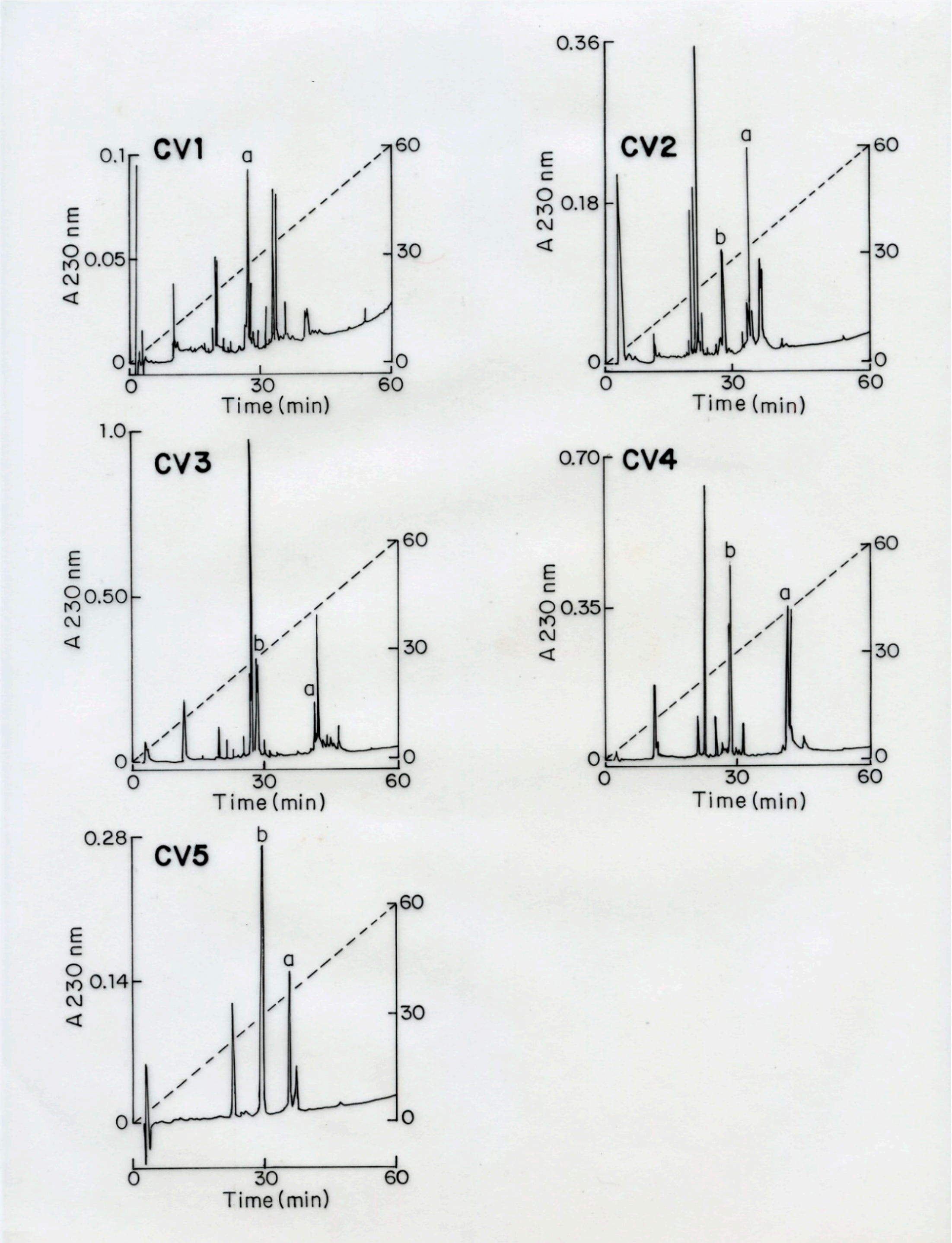

Figure S1.HPLC separation of peptides after enzymatic cleavage. Identical conditions as those reported in Figure 2 were used for separation of the peptides obtained after enzymatic hydrolysis (Asp-N hydrolase) of the 5 purified reduced and alkylated toxins Cv1 to Cv5. Peptides labeled a and b for each one of the toxins were sequenced and the results are shown on Figure 3. For Cv1 the sequence of peptide a, eluted at 26.9 minutes. For Cv2 the peptides b and a eluted at 36.6 and 26.6 minutes respectively. For Cv3 the peptides b and a, eluted at 28.1 and 40.1 minutes, respectively. For Cv4 the peptides b and a eluted ate 28.1 and 40.8 minutes, respectively. For CV5 the peptide b and a eluted at 29.4 and 35.6 minutes

A)

```
                                       10        20        30        40        50        60      Len
                              ....|....|....|....|....|....|....|....|....|....|....|....|....|.
A  C0HMB8|Cv1  |C. villegasi   KEGYIVNLSTGCKYECYKLGDNDYCLKECKLQYGKGAGGYCYAFGCWCTHLYEQAVVWPLPKKTCN    66
B  P45666|Cl11m|C. limpidus    ..........................R...Q...................................T*   66
C  C0HMB9|Cv2  |C. villegasi   .......H..................R.......................................   66
D  Q95WD2|CsE3 |C. sculpturatus .......YH.......F.........R...RH...S...........................K..*  66
E  Q7Z1K8|Cl14 |C. limpidus    .......YHD................R...R................................R..*  66
F  C0HLF3|Co2  |C. ornatus     .......YHD....A.V.........R...R................................R..   66
G  C0HMC2|Cv5  |C. villegasi   .......YYD....A.A.........R...AR...............................K..   66
H  Q9TWL0|Cn8  |C. noxius      .......YY.....FA.A........R...AR...............................K.RA  67
I  C0HMA5|Cbo3 |C. bonito      .......YHD................R...AR..................................   66
J  C0HMA4|Cbo2 |C. bonito      .......YHD................R..RAR..................................   66
K  C0HM18|Chui5|C. huichol     .......YHD................R..R.R............................KN....   66
L  C0HMC1|Cv4  |C. villegasi   .......YHD....A.V.........R..R.R.Y.S........A................N....   66
M  C0HMC0|Cv3  |C. villegasi   .......YYD....A.V.........R..R.R.Y.S........A................N....   66
N  C0HLR5|Cb3  |C. baergi      .......YYD....P.V.........R..R.R.Y.S........A................N...L   66
O  Q7Z1K9|Cl13 |C. limpidus    .......YYD....A.L...E.....R...AR.Y.S........A................N...Y*  66
P  C0HMA6|Cbo4 |C. bonito      ......DYH.....T.A........VR..R.R.YQS.H......A................N.R.K   66
Q  C0HK69|Cl13 |C. limpidus    ....L.DYH.....T.A........VR..R.R.YQS.H......A................N.R.K*  66
R  C0HLR4|Cb2  |C. baergi      ....L.D.H.....T.VG.......VR..R.R.YDS.H.......................N.R.K   66
```

B)

Percent identity between sequences

	A	B	C	D	E	F	G	H	I	J	K	L	M	N	O	P	Q	R
A	100																	
B	95	100																
C	97	95	100															
D	88	86	89	100														
E	91	89	92	92	100													
F	88	86	89	91	97	100												
G	86	86	88	89	92	94	100											
H	84	85	85	87	87	88	94	100										
I	91	91	92	91	97	94	94	88	100									
J	89	89	91	89	95	92	92	87	98	100								
K	88	86	89	88	94	91	88	82	94	95	100							
L	82	80	83	83	88	91	86	81	88	89	91	100						
M	82	80	83	82	86	89	88	82	86	88	89	98	100					
N	80	80	82	80	85	86	85	81	85	86	88	95	97	100				
O	79	80	80	79	83	85	88	84	86	85	83	91	92	91	100			
P	74	74	76	77	80	80	77	76	77	79	80	86	85	85	80	100		
Q	73	73	74	76	79	79	76	75	76	77	79	85	83	83	79	98	100	
R	74	74	74	74	77	79	73	72	74	76	77	82	80	80	74	91	92	100

Percent similarity between sequences

	A	B	C	D	E	F	G	H	I	J	K	L	M	N	O	P	Q	R
A	100																	
B	97	100																
C	98	95	100															
D	91	88	91	100														
E	92	89	92	95	100													
F	89	86	89	92	97	100												
G	89	86	89	91	95	98	100											
H	88	87	88	90	91	94	96	100										
I	94	91	94	94	98	95	94	90	100									
J	94	91	94	94	98	95	94	90	100	100								
K	91	88	91	91	95	92	91	87	97	97	100							
L	86	83	86	86	91	94	92	88	92	92	92	100						
M	86	83	86	85	89	92	94	90	91	91	91	98	100					
N	85	83	85	83	88	89	91	88	89	89	89	95	97	100				
O	85	83	85	83	88	91	92	90	89	89	89	97	98	97	100			
P	82	80	82	85	86	88	86	88	85	85	85	91	89	89	89	100		
Q	82	80	82	85	86	88	86	88	85	85	85	91	89	89	89	100	100	
R	82	80	80	82	83	85	83	85	82	82	82	88	86	86	86	97	97	100

72 — Percent values — 100

Figure S2. Comparison of *C. villegasi* toxin sequences. A) Multiple alignment of toxins from *C. villegasi* and other phylogenetically related β-NaScTxs. Len: indicates mature chain length; Conserved cysteine residues are highlighted in yellow. Positions identical to the *C. villegasi* toxin Cv1 used as a reference are indicated by dots. (*) indicates toxins where C-terminal amidation has been reported. Sequences were ordered according to their position in the phylogenetic tree. B) Matrices with the percentages of identity and similarity between sequences.

```
LR      ■  ▼  ■ ■■■▼■■■■  ■ ■  ■■             ■■■■
Cn2  KEGYLVDKNTGCKYECLKLGDNDYCLRECKQQYGKGAGGYCYAFACWCTHLYEQAIVWPLPNKRCS

Cll1 KEGYIVNLSTGCKYECYKLGDNDYCLRECKQQYGKGAGGYCYAFGCWCTHLYEQAVVWPLPKKTCT
10FG2    ●●●●                     ●●●● ♦                ● ●●●   ● ●


Cv1  ----I-NLS-----E-Y---------K--KLQ-G-G--------G----------V-----K-T-N
Cv2  ----I-NHS-----E-Y---------R--KLQ-G-G--------G----------V-----K-T-N
Cv3  ----I-NYYD----A-V---------R--RLR-Y-S--------A----------V-----N-T-N
Cv4  ----I-NYHD----A-V---------R--RLR-Y-S--------A----------V-----N-T-N
Cv5  ----I-NYYD----A-A---------R--KAR-G-G--------G----------V-----K-K-N
     1       10        20        30        40        50        60
```

Figure S3. Analysis of possible interactions of the evaluated scFvs with *C. villegasi* toxins. Changes between this set of toxins are highlighted in gray. Cn2 is neutralized by the scFv LR whose contacts with the toxin are highlighted with squares (▪) at the top of the sequence. The scFv LR has a very strong interaction with this toxin in the due to the contribution of 3 salt bridges (▼), which are two with D7 and 1 with E15 of the toxin. The contacts of Cll1 with the scFv 10FG2 (●) at the bottom of the sequence are highlighted. The only salt bridge generated is with the K35 of the toxin (♦).
